# PARTICIPATORY ACTION RESEARCH IN GERONTOLOGY: CONCEPTUALIZATION, CONDUCT, AND DISSEMINATION

**DOI:** 10.1093/geroni/igae098.1741

**Published:** 2024-12-31

**Authors:** Jason Flatt, Crystal Glover

**Affiliations:** University of Nevada Las Vegas, Las Vegas, Nevada, United States; Rush University Alzheimer’s Disease Research Center, Chicago, Illinois, United States

## Abstract

Health disparities continue to critically diminish and deleteriously impact health in aging among large diverse populations across the world. One way to create health equity for all older adults remains through the strengths-based sharing of expertise across diverse communities and researchers from various scientific disciplines. As such, the field of Gerontology requires innovative approaches to engage two critical stakeholder groups – community members and researchers – to meaningfully engage and collaborate with each other to conceptualize, conduct, and disseminate related findings stemming from participatory action research (PAR). Resultant research must generate knowledge and actionable solutions that are mutually beneficial, scientifically sound, and culturally compatible across stakeholder groups. In this workshop, we will first teach both researchers and community members about the purpose, process, and intended outcomes of PAR. Second, we will outline the conduct of PAR using a pedagogical framework that includes key topics such as the researcher’s onus of trust, establishing and sustaining relationships, co-learning and co-design, methodological design and data collection, collaborative data analysis and interpretation, dissemination of findings, and planning and implementing evidence- and strengths-based strategic transformations within diverse aging populations. Lastly, we will discuss opportunities and tools for attendees to expand PAR to address health disparities and facilitate health equity in aging among diverse populations. Throughout, we will leverage our extensive PAR experiences to share examples, lived experiences, and best practices with attendees. We hope to host an enriching, relevant, and forward-looking session with attendees.

